# Characterisation of *mycobacteria *isolated from slaughter cattle in pastoral regions of Uganda

**DOI:** 10.1186/1471-2180-7-95

**Published:** 2007-10-25

**Authors:** J Oloya, R Kazwala, A Lund, J Opuda-Asibo, B Demelash, E Skjerve, TB Johansen, B Djønne

**Affiliations:** 1 Department of Veterinary Public Health and Preventive Medicine, Makerere University, Kampala, Uganda; 2 Department of Food Safety and Infection Biology, Norwegian School of Veterinary Science, N-0033, Oslo, Norway; 3 Sokoine University of Agriculture, Morogoro, Tanzania; 4 Department of Animal Health, National Veterinary Institute, N-0033 Oslo, Norway; 5 Department of Veterinary Public Health and Microbiology, University of Hawassa, Awassa, Ethiopia

## Abstract

**Background:**

Bovine tuberculosis is a zoonotic problem in pastoral cattle and communities in Uganda. Tuberculin tests in pastoral cattle had shown a high herd but low animal prevalence, with a high proportion of avian reactors. No work had been done to identify the mycobacterial species involved. The objective of the study was to isolate and characterise Mycobacterial species causing tuberculous lesions in slaughtered animals. Lesioned organs compatible with bovine tuberculosis in slaughtered cattle from pastoral areas in Uganda were collected and cultured to isolate *mycobacteria*. AccuProbe culture identification kits for the *Mycobacterium tuberculosis *complex, *M. avium *complex and *M. avium *were used to identify the isolates. Spoligotyping and Insertion Sequence (IS) *1311 *and IS*1245 *Restriction Fragment Length Polymorphism analysis (RFLP) were used to further characterise the isolates.

**Results:**

Of the 61 lesioned organs and tissues cultured, 19 isolates were identified as *M. bovis*, 3 as *M. avium *subsp.*hominissuis*, 1 as *M. intracellulare*, 1 as a mixed culture of *M. bovis *and *M. avium sp*. and 1 as *M. avium sp*. and unidentified *mycobacteria*. Eleven other *mycobacteria *outside the tuberculosis and avium complex groups were also isolated. Ten new spoligopatterns grouped into three clusters were identified from *M. bovis *isolates. Two of the three *M. avium *subsp.*hominissuis *isolates showed similar patterns on the IS*1311 *RFLP but all were different on the IS*1245 *RFLP.

**Conclusion:**

The isolation of *M. bovis *confirms the ongoing infection with spoligotypes unique to Uganda. Isolation of environmental *mycobacteria *could explain the high avian or non specific tuberculin reactor patterns commonly observed in pastoral cattle and suggests their pathogenic or opportunistic role in the infection of cattle with disseminated bovine tuberculous lesions.

## Background

*Mycobacterium bovis *infections are of major importance in many developed and developing countries, including Uganda [1]. In countries with national bovine tuberculosis eradication programmes, clinical evidence of tuberculosis in cattle is seldom encountered because the intradermal tuberculin test enables presumptive diagnosis and elimination of infected animals before signs appear [2]. Studies have shown that in the final phases of eradication, strains of *Mycobacterium *sp. outside the tuberculosis (MOT) complex may increase the relative frequency and cause false positive skin test reactions [3]. In countries without any control programme, including Uganda, clinical symptoms and post mortem lesions associated with bovine tuberculosis (BTB) may still be seen [4].

Post mortem examination followed by bacteriological examination of suspected lesions in cattle are important tools to confirm the presence[5], while molecular studies help to characterise mycobacterial species involved [6,7]. Histo-pathological examination may increase the specificity of the diagnosis and in circumstances where the prevalence of bovine tuberculosis is high, pathology can be relied on for diagnosis [5]. However, lesions due to an infection with other *mycobacteria *can be mistaken for bovine tuberculosis. When the prevalence is low, as in the latter stages of an eradication campaign or under extensive management systems, the need for definitive diagnosis becomes more important and routine culturing of all suspected lesions is recommended.

The combination of conventional methods and molecular typing of isolates have yielded important insights into the epidemiology of tuberculosis in domestic [8-10] and wildlife populations [11-13]. It has permitted a more precise targeting and monitoring efficacy of conventional control measures [10,14,15]. It has also provided more precise information on the rate of transmission [12,16], stability of the genetic profiles and has been used in epidemiological tracing [9]. A possible relationship exists between the genotype and phenotype of *M. bovis *and the disease pattern [17]. Different genotypes may differ in pathogenicity and thereby affect the development of lesions and how the animals react to the tuberculin test. This suggests a linkage between immunogenicity with respect to the skin test and possibly transmissibility[17], which traditional approaches to tuberculosis diagnosis could not adequately explain.

Different molecular tools have been developed to differentiate between mycobacterial isolates. Notable are spoligotyping and Restriction Fragment Length Polymorphism (RFLP) based molecular techniques[6]. Spoligotyping is designed to detect the presence or absence of unique spacers within the direct repeat (DR) locus of the *M. bovis *genome. It is known to distinguish between phenotypically different strains and has been successfully used to type *M. bovis *isolates from different African countries such as Nigeria[18], Chad [19], Cameroon [20], Madagascar [7] and Tanzania [8]. Spoligotyping has been performed on *M. bovis *isolates obtained from different animals to show the interaction between species. Some spoligotypes have been associated with more sensitivity and specificity to tuberculin, virulence and transmissibility[17].

Different Insertion Sequences (IS)-IS*901*, IS*1311 *and IS*1245 *RFLP have been used to identify and differentiate members outside the tuberculosis complex group [21-25]. IS*901 *RFLPhas been used to establish the relationship between *M. avium *complex strains isolated from birds, animals, humans, and the environment and virulence for poultry [25]. IS*1245 *RFLP has low discriminatory power against *M.a.subsp.avium *but has been very important in the study of epidemiology of *M. a*. subsp.*hominissuis *[23]. A closely related IS element is IS*1311*, which shows 85% sequence identity with IS*1245 *at the DNA level [23]. Molecular typing of MOT is important to identify groups previously thought not to play an important role in human and animal infections.

In Uganda, pastoral communities live in close contact with their cattle. The presence of mycobacterial infections in cattle would represent a huge health risk to their owners. Therefore, in endemic areas where bovine and human tuberculosis coexist, the isolation of *M. bovis *from cattle is important in order to prove its presence and thereby elucidate the risk of spreading the infection to humans [26]. There is little information available on agents causing lesions compatible with tuberculosis in slaughtered cattle in Uganda [1], yet recent studies have shown high herd and low animal prevalence of tuberculin reactors in pastoral areas [27]. The high avian or non-specific tuberculin reactor trends encountered in skin tests in pastoral areas made it necessary to provide information on the species of *mycobacteria *involved.

The aim of the present study was to isolate, identify and characterise mycobacterial species causing pathological lesions compatible with bovine tuberculosis in slaughtered cattle from pastoral areas in Uganda.

## Results

**Figure 1:**
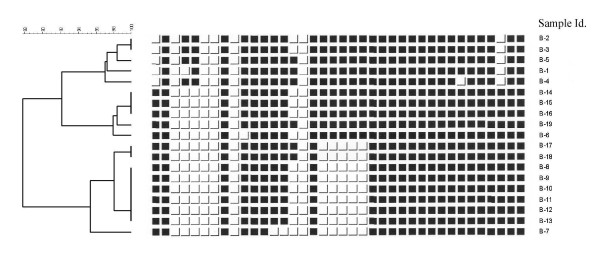
Spoligotypes of *M. bovis *isolated from slaughtered cattle in Uganda. Lack of spacers 39–43 is not shown in the figure.

Thirty seven mycobacterial isolates were cultured from 61 samples collected from organs showing lesions compatible with bovine tuberculosis (Table 1). MTC was detected in 20 of the samples; 15 isolates originated from tuberculous lesions (carcase and lung) and five from localised lymph node lesions. Due to the contamination of subcultures, only fifteen *M. bovis *isolates from this study and four additional *M. bovis *isolates collected from a parallel field study in cattle in the same areas were examined by spoligotyping, ten distinct spoligo patterns were identified. They were grouped into three clusters of >95% similarity (Figure 1). All isolates lacked spacers 3, 9, 16 and 39–43 as most *M. bovis *isolates do and they also lacked spacers 6 and 7. In addition, members of the first cluster (B-1, B-2, B-3, B-4 and B-5) lacked spacers 1 and 36. They showed slight variations, as B-1 lacked spacer 4, B-2 and B-3 lacked spacer 15 and B4 lacked spacer 32. All members of the second cluster lacked spacers 4 and 5, all except one (B-19) lacked spacer 15 and B-6 lacked spacer 10. The nine members of the third cluster lacked spacers 4, 5 and 18–22. In addition B-7, B-8, B-9, B-10, B-11, B-12 and B-13 lacked spacers 15, while B-7 also lacked spacers 13 and 14. The samples were too few to establish any association between the spoligotypes and nature of the lesions they were isolated from.

**Table 1:** *Mycobacteria *isolated from 61 tuberculous lesions in pastoral slaughtered cattle in Uganda

**Mycobacterial species**	**Number**	**Percent**
*M. bovis*	19	51.4
*M. avium *subsp *. hominissuis*	3	8.1
*M. intracellulare*	1	2.7
*M. avium sp*.	1	2.7
*M. bovis + M. avium sp*.	1	2.7
*M*. avium sp. + unidentified *mycobacteria*	1	2.7
Unidentified *mycobacteria*	11	29.7
Total	37	100.0

**Figure 2:**
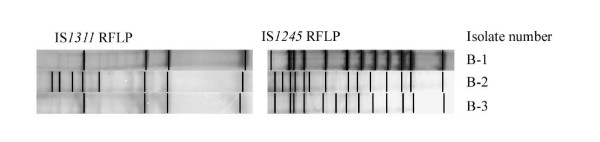
The IS*1311 *and *1245 *RFLP patterns of *M. avium sp hominissuis*.

MOT were detected in 18 samples. Of these, one isolate was identified on MAC and *M. avium *accuprobes as *M. intracellulare *and two others as *M. avium *sp. Most of the unidentified *mycobacteria *(10 out of 11) were isolated from disseminated tuberculous lesions and only one was from a local lymph node (lateral retropharyngeal). The two cases of mixed infections were isolated from disseminated tuberculosis lesions. Of the three *M. avium sp*. examined by IS*1245 *and IS*1311 *RFLP, two showed four bands on the IS*1311 *RFLP while the last isolate showed 8 bands. All three isolates showed different patterns with 13 or 14 bands on IS*1245 *RFLP and were identified as M. *avium *subsp. *hominissuis *[22,28] (Figure 2).

## Discussion

Different mycobacterial species were isolated in lesions compatible with bovine tuberculosis in pastoral slaughtered cattle in Uganda. *M. bovis *was found in 51.4% and *mycobacteria *outside the *M. tuberculosis *complex (MOT) in 48.6% of the 37 samples where *mycobacteria *were detected. These results were comparable to the 42% isolation rate of *M. bovis *in *mycobacteria *positive cultures in Chad [19]. This is the first study in Uganda to document that other mycobacterial species are involved in cattle infections and also reports the spoligotypes of *M. bovis *involved. Earlier identification studies were mainly based on colonial and biochemical characteristics [1].

Many thousands of cattle were examined during the nine month study period, still only 61 animals with macroscopic lesions compatible with bovine tuberculosis were detected. The low number of lesioned animals reflect the low prevalence of BTB in Uganda [27] and probably also the low sensitivity of post mortem examinations [1,5]. However, the isolation of *M. bovis *from slaughtered cattle confirms that bovine tuberculosis is present in pastoral areas in the cattle corridor of mid-central and mid-western regions of Uganda. As documented elsewhere in Africa [29], pastoralists in Africa in general have an obsessive attachment to their cattle. Their diet mostly of raw milk, fresh blood and occasionally meat could be regarded as potential sources of infection [30]. The isolation of *M. bovis *from their cattle should be considered a serious public health concern [31]. In endemic areas where bovine and human tuberculosis coexist, the documentation of the existing strains of *M. bovis *is important for monitoring the spread of *M. bovis *among cattle and between cattle and humans [26]. Two of the *M. bovis *spoligotypes (B-6 and B-13) isolated from slaughtered cattle were also isolated from 3 human cases of cervical lymphadenitis, in a parallel study in humans, though from different locations [32]. This documents an existing infection from cattle to humans and further highlights the potential threat of BTB to humans in rural communities [26,33-35].

Owing to the difficulties encountered in tracking the origin of these cattle, it was not possible to link them to any specific geographic area. However, spoligotypes found in other African countries [7,8,18,20] were not detected in Uganda. Comparison of the Ugandan spoligotypes with the *M. bovis *database [49] and tuberculosis database SpolDB4 [36] showed that these particular spoligotypes were unique and not documented elsewhere.

Isolation of *M. avium *subsp. *hominissuis *and other MOT in cattle with disseminated tuberculosis lesions could signify the importance of these bacteria in pastoral communities and their cattle. *M. avium sp. hominissuis *and other environmental *mycobacteria *have been isolated from swine, cattle, sheep, goats and horses [25,37], wild and domestic birds [3] and humans [38-40]. In contrast to *M. avium *subsp. *avium *infections in wild and domestic birds, these infections in mammals occur only sporadically and are rarely transmissible [3]. Generalised disease due to MOT is usually uncommon, owing to the non-progressive, chronic character of the infection. However, some cases of disseminated disease have been reported, especially in captive non-domestic hoofed animals and pigs [41]. The relatively high rate of isolation of MOT (48.6%) in the current study (Table 1), calls for special attention. The isolation of *mycobacteria *mostly considered opportunistic or less virulent [3,42] in conditions of disseminated tuberculosis with fully caseated and calcified lesions is documented elsewhere[3]. However, mixed infections, as documented in other studies [43], were observed in caseated and/or calcified disseminated lesions in this study. These findings could suggest secondary mycobacterial infections or may have outgrown the slow growing *M. bovis *during culture[3]. Isolation of MOT in this study is consistent with earlier findings in pastoral areas, in which high avian and doubtful reactor trends were reported [27]. Sensitisation of test animals with environmental *mycobacteria *have been documented as interfering with the interpretation of tuberculin results by affecting sensitivity and specificity [44].

Many mycobacterial species are ubiquitous organisms in environmental reservoirs; especially in water and soil [3,42,45-47] and can survive and multiply under a wide range of environmental conditions. Their recovery from tissues of slaughter animals originating from such areas is indicative of the mycobacterial status of the environment and water sources [3,45,46] as well as their potential to cause infection [3]. In most pastoral areas in Uganda, animals drink from natural water holes, dug out ponds or valley dams or tanks, which are often heavily contaminated with animal waste. As documented, water high in organic matter or animal dejections enhances the growth of environmental *mycobacteria *[3] and such water constitutes the greatest risk of exposure to humans and animals [42,48].

## Conclusion

This study confirmed the presence of *M. bovis *among cattle in the pastoral cattle corridor in mid-central and mid-western areas of Uganda and detected new and specific spoligotypes not reported before. Furthermore, the study gives an insight into other mycobacterial species infecting cattle in this region and thereby contributes to explaining the high avian or non specific reactor patterns commonly observed in tuberculin tested animals in these areas. Strong avian reactions in the tuberculin testing scheme reduce the tuberculin test specificity, something that calls for a review of the interpretation of tuberculin tests in pastoral areas.

## Methods

### Collection of samples

The samples were collected from slaughtered cattle originating from pastoral areas in the cattle corridors of Nakasongola, Luwero, Masindi and Mbarara districts brought to Kalerwe Abattoir, North of Kampala during a nine month period between 2004 and 2005. It was not possible to trace the place of origin of the animals, due to poor documentation, cattle markets with large overlapping catchment areas spanning many districts and the criss-crossing nature of rural transportation of animals and traders withholding information due to the illegal movement of cattle in areas under quarantine. Tissue samples from 61 cattle showing macroscopic lesions compatible with bovine tuberculosis during the post mortem examination were collected and submitted to the laboratory for processing and storage at -70°C.

### Tissue Preparation and Culture

Fat and connective tissue were removed from the specimens and approximately 10 g were placed in sterile stomacher bags containing 30 ml of physiological buffered saline and homogenised in the stomacher machine for 7–10 minutes. The homogenate was transferred into tubes and an equal volume of Sodium hydroxide-N-acetyl-L-cysteine (NaOH-NALC) decontamination solution [containing 6.8% Sodium Hydroxide (NaOH), 2.9% sodium citrate and 0.5% N-acetyl-L-cysteine (NALC) to a final concentration of 1.7% NaOH] was added. After vortexing, the specimens were left at room temperature for 15 min. Subsequently 5 ml of sterile 0.067 M phosphate buffer (pH 6.8) was added and the mixture was centrifuged at 3660 × g for 15 min. The supernatant was discarded and the sediment was inoculated on Lowenstein-Jensen (Difco Laboratories, Detroit, Michigan) media with and without pyruvate (0.6%) and incubated at 37°C. Tubes were read weekly for up to 12 weeks. Of the 61 lesioned specimens collected and cultured, 37 grew *mycobacteria*. Four additional mycobacterial isolates were obtained from apparently normal lymph nodes from a parallel study in the same area. Typical or suspect colonies were harvested into cryotubes containing 1.5 ml Middlebrook 7H9 (Difco Laboratories, Detroit, MI) and stored at -70°C at the National Tuberculosis Referral Laboratory, Uganda. The isolates were transported to the National Veterinary Institute, Oslo and sub cultured on Middlebrook 7H10 and Stonebrink media (Difco Laboratories, Detroit, MI).

### Identification of mycobacterial isolates

All acid fast bacteria, determined by the Ziehl-Neelsen (ZN) staining technique, were examined by the AccuProbe MTC (mycobacterium tuberculosis complex) identification kit (GEN-PROBE^® ^INC. San Diego, California) as described by the producer. Results were considered positive when Relative Light Units (RLU) were greater than 30 000, repeat range; 20 000–29 000 and negative; below 20 000. Samples negative on the MTC kit were examined further on the AccuProbe MAC (*M. avium *complex) and AccuProbe *M. avium *culture identification kits. Final results were interpreted as follows; cultures positive on the *M. avium *and MAC culture identification kits were considered *M. avium *species and cultures negative on the *M. avium *kit and positive on the MAC kit were identified as *M. intracellulare*. Samples negative on all AccuProbe culture identification kits were grouped as unidentified *mycobacteria*.

### Spoligotyping and Analysis of the spoligotyping patterns

Cultures belonging to the *M. tuberculosis *complex were submitted for further differentiation by Spoligotyping [20] as described by the producer (Isogen, Life science, The Netherlands). DNA was isolated using theQuiagen DNA extraction Kit for cell cultures (Qiagen™, Nordic) following the manufacturer's instruction manual, PCR and hybridisation were performed as described [21]. The amplified DNA was hybridised to a membrane containing 43 oligonucleotides in a miniblotter (MN45, Immunetics, Cambridge, Massachusetts). Bound fragments were revealed by chemiluminescence after incubation with horseradish peroxidase-streptavidin (Boehringer, Mannheim, Germany) for 45 min at 45°C and the membrane was exposed to X-ray film (Hyperfilm, Amersham) for 10–12 min. The *M. tuberculosis *H37Rv and *M. bovis *BCG were included as controls.

The results were analysed using the BioNumerics programme version3.5(Applied Maths, Kortrijk, Belgium). Normalisation of the fingerprints was done using the molecular weight standard 1 kb DNALadder (Invitrogen™, Carlsbad, Calif.). The BioNumerics software was used to calculate Dice coefficients of similarity and to cluster the isolates and generate dendograms by the unweighted-pair group method using linkage averages. The optimisation and tolerance settings were both set at2.00%.

### Examination by IS*1311 *and IS*1245 *RFLP

Cultures identified as *M. avium sp*. were further examined using IS*1245 *and IS*1311 *Restriction Fragment Length Polymorphism (RFLP). The DNA extraction, RFLP analysis and interpretation of results were performed as previously described [22], using the described probes for IS*1245 *and IS*1311 *RFLP.

## Competing interests

The author(s) declares that there are no competing interests.

## Authors' contributions

JO: Principal investigator, participated in the conception and design of the study, general conduct of the study, acquisition of data, isolation and culture of *mycobacteria*, molecular studies and drafted manuscript. R.K: Conception and design of the study, general conduct of the study, acquisition of data, isolation and culture of *mycobacteria*. AL: Participated in the conception and design of the study, acquisition of funding, general conduct of the study, isolation and culture of *mycobacteria *and critical revision of the manuscript for important intellectual content. J.O-A: Participated in the conception and design of the study, general supervision of the research group in Uganda. General conduct of the study, acquisition of data, isolation and culture of *mycobacteria*, molecular studies and critical revision of the manuscript for important intellectual content. ES:Acquisition of funding, general supervision of the research group in Norway, participated in the conception and design of the study, acquisition of data, drafted the manuscript. B.D: Participated in the design of the study, general supervision of the laboratoty work in Norway, isolation and culture, molecular studies and critical revision of the manuscript for important intellectual content. TBJ: Carried out the molecular genetic studies, participated in the sequence alignment and drafted the manuscript. BD:Isolation and culture, molecular genetic studies and critical revision of the manuscript for important intellectual content.

All the authors have read and approved the final version of the manuscript.
